# Supplementary material for: High expression of olfactomedin-4 is correlated with chemoresistance and poor prognosis in pancreatic cancer
Source: PLoS One. 2020 Jan 10;15(1):e0226707. doi: 10.1371/journal.pone.0226707 (PMC6953839; doi:10.1371/journal.pone.0226707)
Supplement: S1 Fig — (PPTX) [file pone.0226707.s002.pptx]

## Slide 1
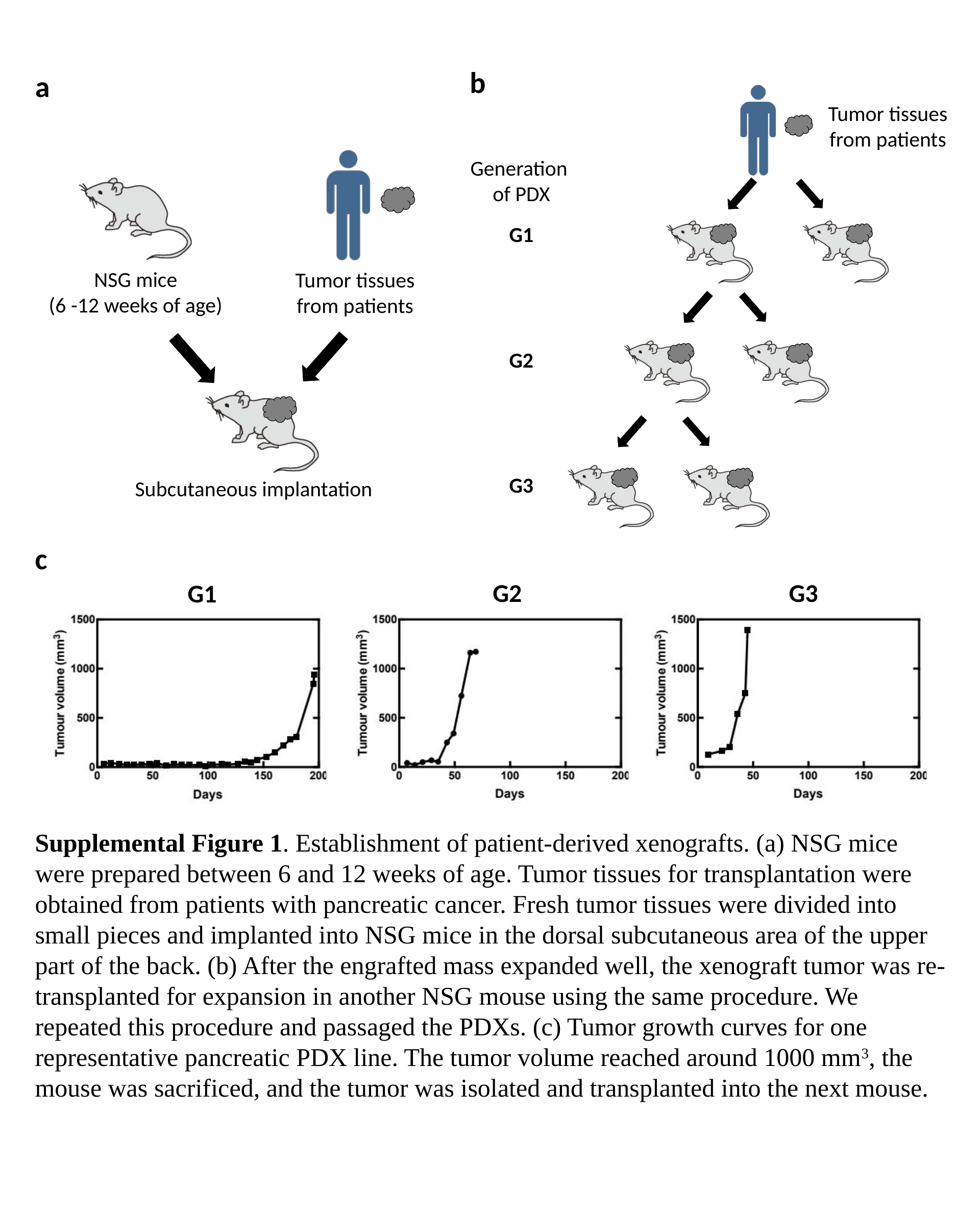

b
a
Tumor tissues from patients
Generation
of PDX
G1
G2
G3
NSG mice
(6 -12 weeks of age)
Tumor tissues
from patients
Subcutaneous implantation
G3
G2
G1
c
Supplemental Figure 1. Establishment of patient-derived xenografts. (a) NSG mice were prepared between 6 and 12 weeks of age. Tumor tissues for transplantation were obtained from patients with pancreatic cancer. Fresh tumor tissues were divided into small pieces and implanted into NSG mice in the dorsal subcutaneous area of the upper part of the back. (b) After the engrafted mass expanded well, the xenograft tumor was re-transplanted for expansion in another NSG mouse using the same procedure. We repeated this procedure and passaged the PDXs. (c) Tumor growth curves for one representative pancreatic PDX line. The tumor volume reached around 1000 mm3, the mouse was sacrificed, and the tumor was isolated and transplanted into the next mouse.
