# Supplementary material for: High expression of olfactomedin-4 is correlated with chemoresistance and poor prognosis in pancreatic cancer
Source: PLoS One. 2020 Jan 10;15(1):e0226707. doi: 10.1371/journal.pone.0226707 (PMC6953839; doi:10.1371/journal.pone.0226707)
Supplement: S2 Fig — (PPTX) [file pone.0226707.s003.pptx]

## Slide 1
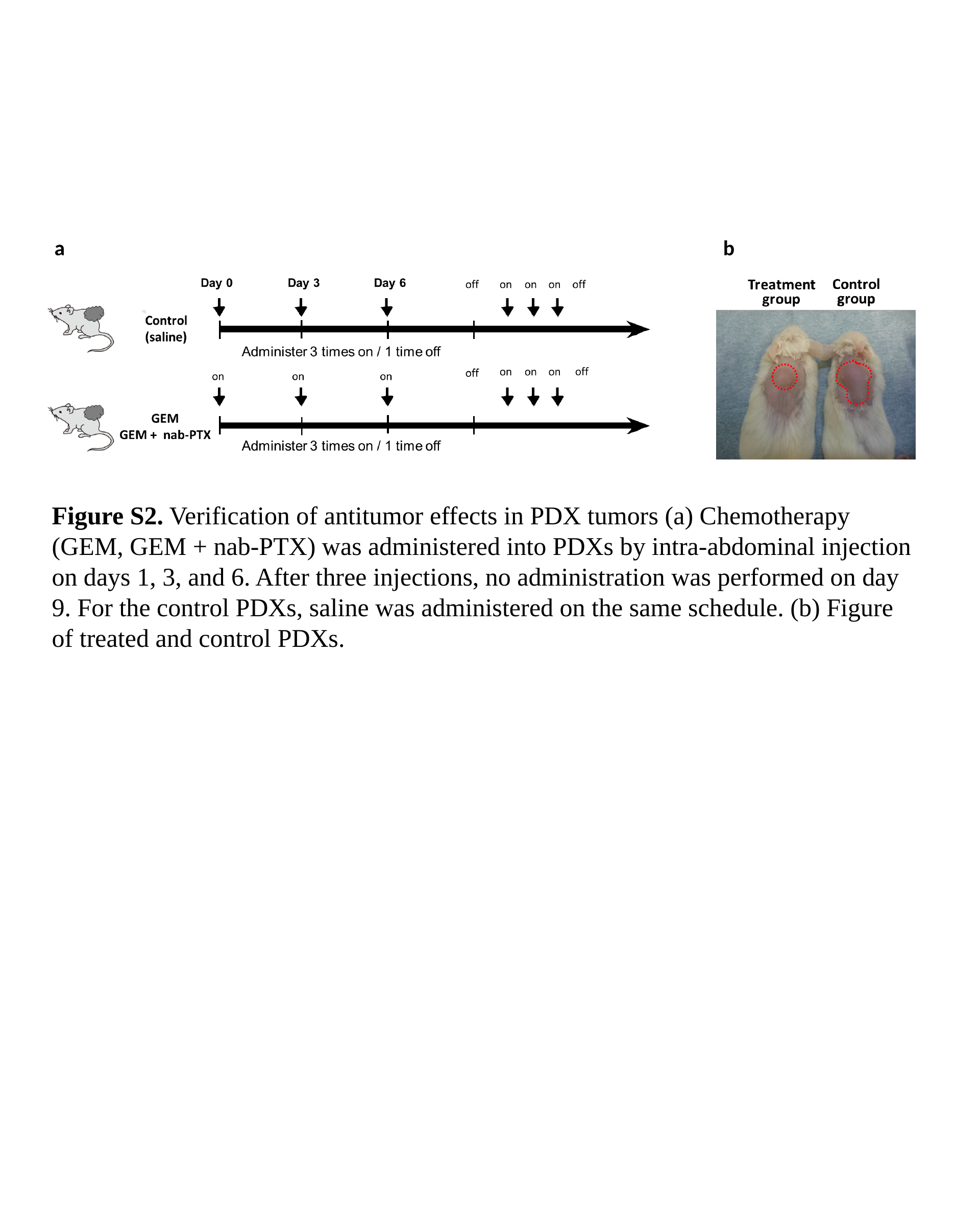

a
b
e
Figure S2. Verification of antitumor effects in PDX tumors (a) Chemotherapy (GEM, GEM + nab-PTX) was administered into PDXs by intra-abdominal injection on days 1, 3, and 6. After three injections, no administration was performed on day 9. For the control PDXs, saline was administered on the same schedule. (b) Figure of treated and control PDXs.
