# Supplementary material for: High expression of olfactomedin-4 is correlated with chemoresistance and poor prognosis in pancreatic cancer
Source: PLoS One. 2020 Jan 10;15(1):e0226707. doi: 10.1371/journal.pone.0226707 (PMC6953839; doi:10.1371/journal.pone.0226707)
Supplement: S3 Fig — (PPTX) [file pone.0226707.s004.pptx]

## Slide 1
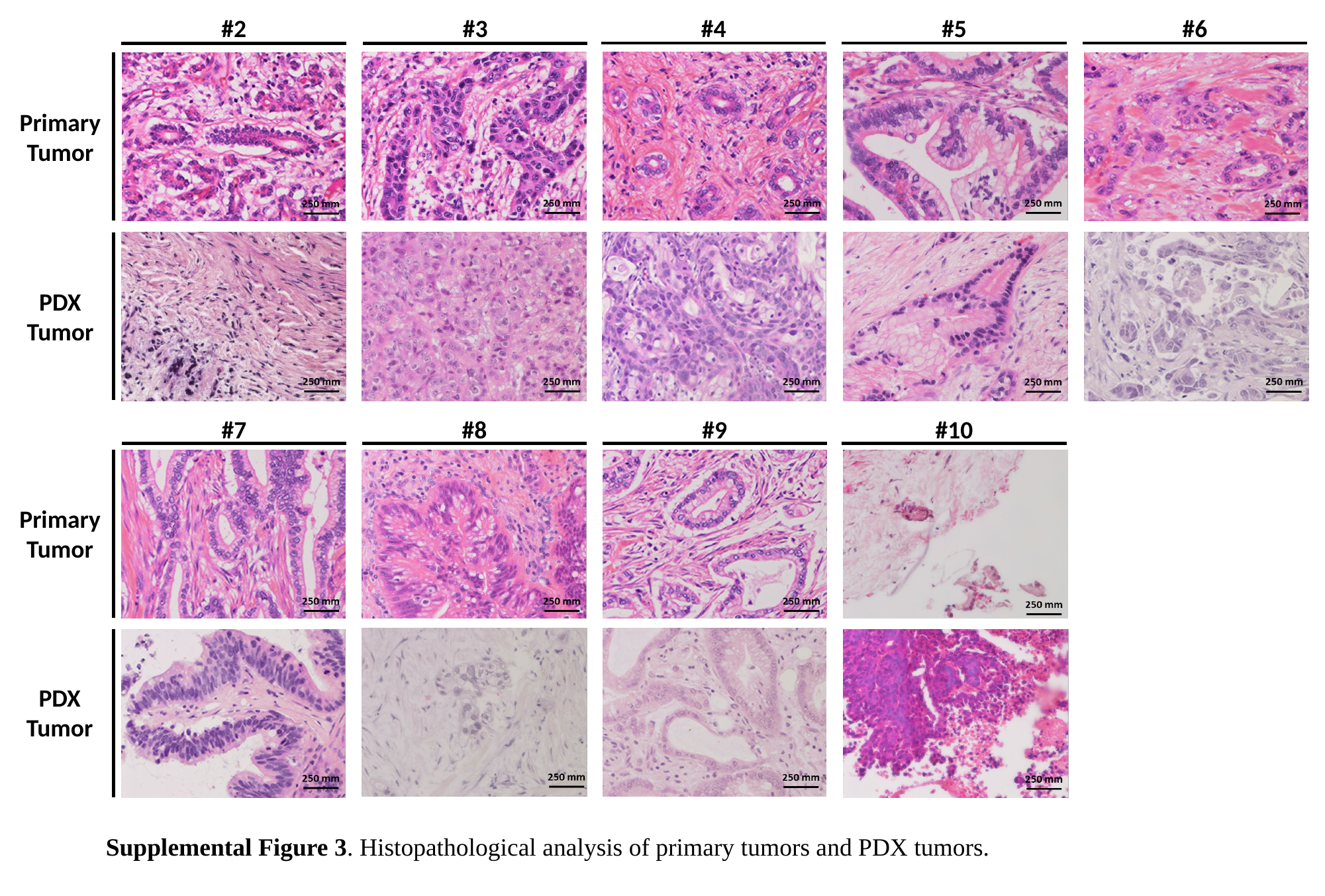

#4
#5
#6
#2
#3
Primary Tumor
PDX Tumor
#7
#8
#9
#10
Primary Tumor
PDX Tumor
Supplemental Figure 3. Histopathological analysis of primary tumors and PDX tumors.
