# Supplementary material for: High expression of olfactomedin-4 is correlated with chemoresistance and poor prognosis in pancreatic cancer
Source: PLoS One. 2020 Jan 10;15(1):e0226707. doi: 10.1371/journal.pone.0226707 (PMC6953839; doi:10.1371/journal.pone.0226707)
Supplement: S4 Fig — (PPTX) [file pone.0226707.s005.pptx]

## Slide 1
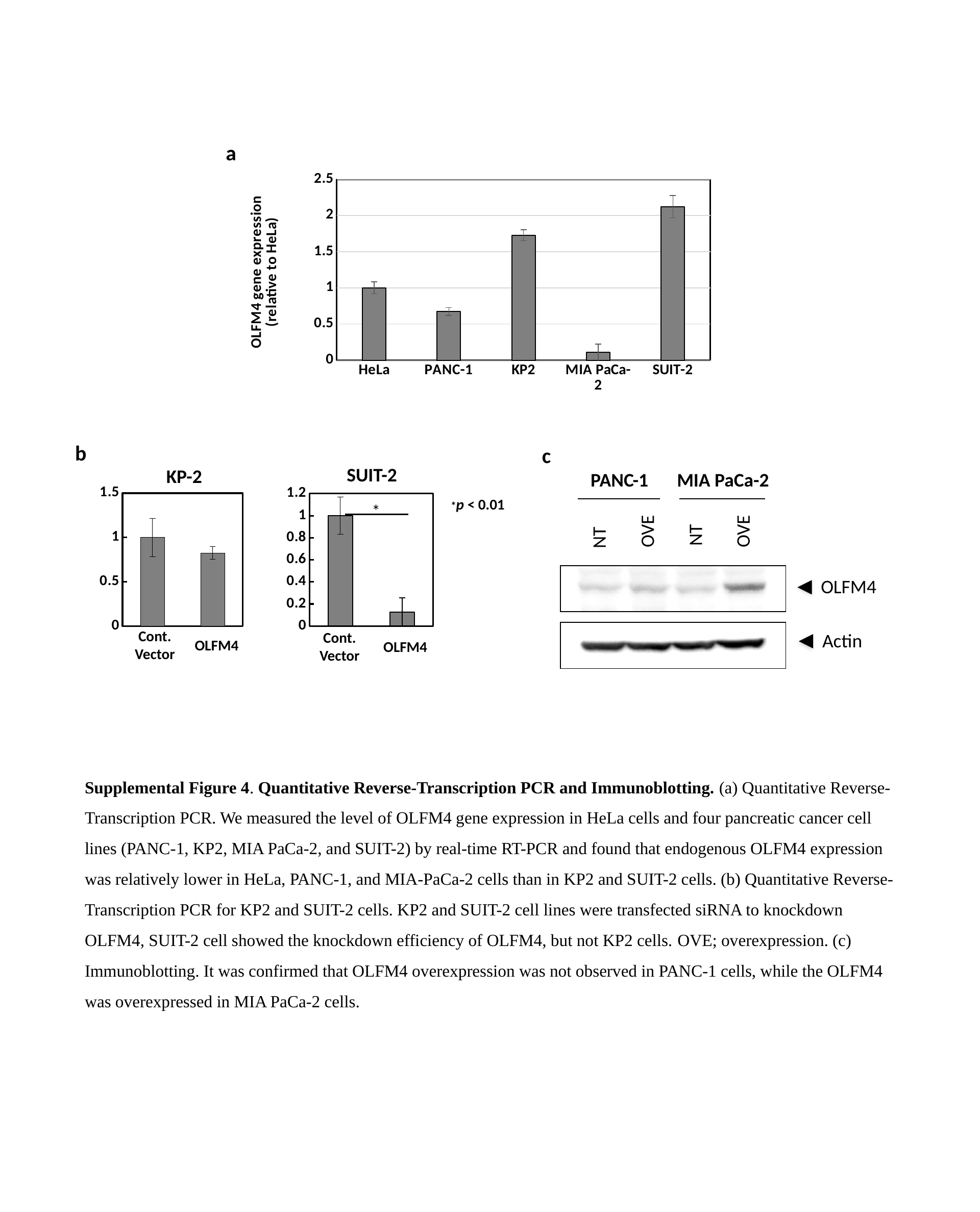

a
### Chart
| Category | |
|---|---|
| HeLa | 1.0 |
| PANC-1 | 0.672328 |
| KP2 | 1.727353 |
| MIA PaCa-2 | 0.109102 |
| SUIT-2 | 2.122281 |b
c
SUIT-2
### Chart
| Category | |
|---|---|
| SUIT-2_Ctrl Vector | 1.0 |
| SUIT-2_OLFM4 | 0.12773069886332 |Cont. Vector
OLFM4
KP-2
### Chart
| Category | |
|---|---|
| KP-2_Ctrl Vector | 1.0 |
| KP-2_OLFM4 | 0.826483141968719 |Cont. Vector
OLFM4
PANC-1
MIA PaCa-2
OVE
OVE
NT
NT
OLFM4
Actin
*p < 0.01
*
Supplemental Figure 4. Quantitative Reverse-Transcription PCR and Immunoblotting. (a) Quantitative Reverse-Transcription PCR. We measured the level of OLFM4 gene expression in HeLa cells and four pancreatic cancer cell lines (PANC-1, KP2, MIA PaCa-2, and SUIT-2) by real-time RT-PCR and found that endogenous OLFM4 expression was relatively lower in HeLa, PANC-1, and MIA-PaCa-2 cells than in KP2 and SUIT-2 cells. (b) Quantitative Reverse-Transcription PCR for KP2 and SUIT-2 cells. KP2 and SUIT-2 cell lines were transfected siRNA to knockdown OLFM4, SUIT-2 cell showed the knockdown efficiency of OLFM4, but not KP2 cells. OVE; overexpression. (c) Immunoblotting. It was confirmed that OLFM4 overexpression was not observed in PANC-1 cells, while the OLFM4 was overexpressed in MIA PaCa-2 cells.
