# Supplementary material for: High expression of olfactomedin-4 is correlated with chemoresistance and poor prognosis in pancreatic cancer
Source: PLoS One. 2020 Jan 10;15(1):e0226707. doi: 10.1371/journal.pone.0226707 (PMC6953839; doi:10.1371/journal.pone.0226707)
Supplement: S1 Text — (DOC) [file pone.0226707.s006.doc]

*Supplementary Information*

*Material & Methods for Supplemental Figure 4*

**Quantitative Reverse-Transcription PCR**

Total RNA was prepared using a RNeasy mini kit (Qiagen, Hilden, Germany) according to the manufacturer’s procedure and reverse transcribed using random primers and Superscript IV VILO (Invitrogen, Carlsbad, CA, USA) according to the manufacturer’s instructions. cDNA was amplified by real-time PCR System (StepOnePlus Real-Time PCR System, Applied Biosystems, St. Austin, TX, USA) with TaqMan Fast Advanced Master Mix (Applied Biosystems) to quantify expression of OLFM4 and ACTB mRNA. The specific TaqMan probes were: OLFM4, Hs00197437_m1; ACTB, Hs01060665_g1 (Applied Biosystems).

**Immunoblotting**

Whole cell lysates were prepared in RIPA buffer (Nacalai tesque, Kyoto, JPN) with Complete Mini Protease Inhibitor Cocktail (Roche, Basel, Switzeland) and phosphatase inhibitor cocktail (PhosSTOP, Sigma Aldrich). Whole cell extracts (100 μg protein) were separated by SDS-PAGE using an XCell SureLock Mini-Cell with Bis-Tris gels in MOPS SDS running buffer (Invitrogen) and transferred to nitrocellulose membranes (Bio-Rad, Hercules, CA, USA), according to the manufacturer’s instructions. Samples were blocked with 5% bovine serum albumin in Tris-buffered saline with 0.1% Tween (TBST). Membranes were then probed overnight at 4 ◦C with the following primary antibodies diluted in TBST/5% BSA: monoclonal anti-GFP (1E4, Medical & Biological Laboratories; 1:1000, Nagoya, JPN), monoclonal anti-β-actin (AC-15, Sigma; 1:4000, St. Louis, MO, USA), Membranes were washed in TBST and probed with horseradish peroxidase-coupled anti-mouse secondary antibodies (Cell Signaling Technology, Danvers, MA, USA) diluted 1:2000 in TBST/5% Difco Skim Milk (Becton, Dickinson and Company, MD, USA) for 1 h. Finally, the membranes were washed and developed with ECL Western Blotting Detection Reagents (GE Healthcare, Chicago, IL, USA). Signal intensities were detected with a ChemiDoc XRS+ and Image lab Software (GE Healthcare).
