# Supplementary material for: High expression of olfactomedin-4 is correlated with chemoresistance and poor prognosis in pancreatic cancer
Source: PLoS One. 2020 Jan 10;15(1):e0226707. doi: 10.1371/journal.pone.0226707 (PMC6953839; doi:10.1371/journal.pone.0226707)
Supplement: Attachment — Submitted filename: Response to Reviewers_2.docx [file pone.0226707.s007.docx]

November 14, 2019

Dr. Masaru Katoh

Academic Editor

PLOS ONE

U.S. Headquarters Public Library of Science

1160 Battery Street, Koshland Building East, Suite 100

San Francisco, CA 94111 United States

Re: Manuscript ID: PONE-D-19-18437

Dear Dr. Masaru Katoh,

Thank you very much for your e-mail and review of the manuscript (PONE-D-19-18437) that we sent on Aug 3, 2019. We are grateful to you and the reviewer for your comments and suggestions, which have helped us to improve our manuscript. We have revised the manuscript based on these comments and have provided our point-by-point responses to each of the comments below. We have carried out additional experiments, including the investigation for OLFM4 overexpression and knockdown experiments using pancreatic cancer cell lines, in response to the reviewers’ major criticisms. Newly generated data reinforce the function of OLFM4 as a chemotherapy (gemcitabine) resistant factor.

We have included our revised manuscript. All changes have been made in response to the editor’s and reviewer’s suggestions, and an itemized response to the individual reviewer’s comments is also attached. We have also updated the manuscript according to journal requirements.

**Response to the Editor’s and Reviewer’s Comments**

**Editor:**

(1) Next-generation sequencing (NGS). You need to carry out whole-exome sequencing and analyze genetic alterations to investigate the mechanisms of chemoresistance in pancreatic cancer patients.

Answer:

We sincerely appreciate your comment.

We performed next-generation sequencing (NGS) of tumor tissues from PDXs before and after treatment with standard chemotherapy. Regarding Figure 2, in the Gemcitabine administration group, the number of gene mutations (missense mutations) after chemotherapy administration compared to before treatment was 1 (PDX #1), 1 (#3), 1 (#4), 11 (#5), 2 (#7) and 2 (#9). For the Gemcitabine + nab-Paclitaxel administration group, the number of gene mutations after chemotherapy administration was 4 (#1), 7 (#2), 1 (#3), 0 (#4), 2 (#5), 1 (#6), 1 (#7), 6 (#8), 1 (#9), and 0 (#10). Therefore, the number of secondary gene mutations was less than expected due to the administration of anticancer drugs. All secondary gene mutations were of the passenger type, and no driver mutations were detected. Furthermore, no common gene mutations were found between chemotherapy groups. From the above, we concluded that although chemoresistance was not due to genetic mutations after chemotherapy administration, *OLFM4* mRNA expression was affected. We appreciate your helpful comment, and have added an explanation to the discussion section (P27-28, lines 452-457).

(2) OLFM4 overexpression experiments in HeLa cells. You should describe how you established HeLa cells overexpressing OLFM4.

Answer:

Thank you for your helpful comment. HeLa cells were transfected with plasmid containing DNA of interest using FuGENE 6 Transfection Reagent (Progema, Madison, WI, USA), following the manufacturer’s protocol. For the OLFM4 transfection assay, HeLa cells were seeded in a 6-well Falcon plate. After 24 hours, cells were transfected with pCMV6-Ac-*OLFM4*-GFP tag plasmids (NM_006418, OriGene Technologies, Inc., Rockville, MD, USA) and an empty vector as a control, according to the manufacturer’s protocol. As indicated, we have included an explanation for how to establish HeLa cells overexpressing *OLFM4* in the Material and Methods section (P13, lines 204-211).

(3) OLFM4 overexpression and knockdown experiments in pancreatic cancer cells. You need to investigate OLFM4 expression levels in pancreatic cancer cell lines, and then carry out OLFM4 overexpression experiments in pancreatic cancer cell lines with endogenous OLFM4 downregulation, and also OLFM4 knockdown experiments in pancreatic cancer cell lines with endogenous OLFM4 upregulation.

Answer:

　We sincerely appreciate your comment. According to your helpful comment, we carried out the additional experiments for using pancreatic cancer cell lines.

We investigated the assay of OLFM4 expression levels in pancreatic cancer cell lines, such as PANC-1, KP2, MIA PaCa-2 and SUIT-2 cell line. In addition, we demonstrated the cell viability assay for Gemcitabine addition in PANC-1 and MIA PaCa-2 cell line with endogenous OLFM4 downregulation, and in KP2 and SUIT-2 cell line with endogenous OLFM4 upregulation.

PANC-1 and MIA PaCa-2 cells were transfected pCMV-6-OLFM4-GFP and an empty vector, then it was confirmed OLFM4 overexpressed in only MIA PaCa-2 cells. Then we performed the cell viability assay for MIA PaCa-2 cells, the OLFM4 overexpressing cells were more remained than the control cells after adding Gemcitabine. Additionally, KP2 and SUIT-2 cells were transfected siRNA targeting OLFM4. The OLFM4 expression was knockdown in SUIT-2 cell lines, but OLFM4 knockdown was not observed in KP2 cell lines. We performed the cell viability assay for SUIT-2 cells, the OLFM4 knockdown cells were less viable than the control cells after adding Gemcitabine. From these results, we demonstrated the chemoresistant role for OLFM4 expression even in pancreatic cancer cell lines both with endogenous OLFM4 downregulation and upregulation.

As indicated, we have changed the Figure 7b-d (Fig 7) and the Figure Legends (P23, lines 393-399). We have included an explanation for the additional experiment in the section of Material and Methods (P12-14, lines 193-221) and Results (P22-23, lines 377-391). Supplementary Figure 4 (S4 Fig) was newly created to explain the results of the additional experiments. And based on the results of additional experiments, we have modified the part of Discussion section (P28, lines 459-462).

(4) Pancreatic cancer PDXs. You are advised to describe success rate of PDX establishment in your group.

Answer:

Thank you for your helpful comment. We established 61 PDX lines from 116 surgically removed tumor tissues inoculated subcutaneously into NSG mice at the Kanagawa Cancer Center [Chijiwa T, et al. Int J Oncol. 2015;47: 61-70]. The success rate was 53%. Regarding PDX for pancreatic cancer, 10 PDX lines were established from 19 surgically removed pancreatic cancer tumor tissues, and the success rate was 52.6%. As indicated, we have added an explanation for the PDX establishment success rate in the Discussion section (P29, lines 477-482).

(5) Histochemical analyses. You showed histochemical analysis of primary tumor and PDXs in one case. You need to show results for other cases in a supplementary Figure.

Answer:

　Thank you for your helpful comment. Since HE staining was performed in all cases, we presented them as a supplementary Figure 3 (S3 Fig). In nine cases other than Figure 1a, the histopathological characteristics of the patient tumor tissues matched that of PDX models. We appreciate your helpful comment, and have added an explanation to the section of Results (P17, lines 291-292).

**Reviewer #1:**

Ohkuma and colleagues reported that high expression of OLFM4 was involved in chemoresistance and was an independent prognostic factor in pancreatic cancer. The study is interesting and the manuscript is well-written. My only concern is that the discussion is mostly covered by the repetition of the results and should be improved. The authors should explain the similarities and differences between the current study and published literature.

-Wang XY, et al. World J Gastroenterol. 2018;24(17):1881-1887.

-Guette C, et al. Proteomics Clin Appl. 2015;9(1-2):58-63.

-Yan H, et al. Hepatogastroenterology. 2011;58(109):1354-9.

Answer:

We sincerely appreciate your helpful comment. We partially modified the discussion section to explain the similarities and differences between recent studies and our study.

　 Several studies have investigated the role of OLFM4 in tumor differentiation [Guette C, et al. Proteomics Clin Appl. 2015;9(1-2):58-6]. Increased expression of OLFM4 has been found to correlate with tumor differentiation state in gastric and cervical cancers, as determined through IHC analysis. In our study, the expression of OLFM4 did not differ according to degree of tumor differentiation.

　OLFM4 expression has been reported in several solid cancers and has been shown to be predictive of prognosis. One study found that OLFM4 independently predicted a poor prognosis in ER-positive breast cancer [Mayama et al. Cancer Sci. 2018;109: 3350-3359]. On the other hand, OLFM4 is correlated with a better prognosis in gastric and colorectal cancers [Wang XY, et al. World J Gastroenterol. 2018;24(17):1881-1887]. Therefore, the correlation between OLFM4 expression and cancer prognosis is controversial and has not yet been confirmed. In our study, we demonstrated that high expression of OLFM4 was significantly associated with poor prognosis in pancreatic cancer through IHC analysis.

　Yan H et al. demonstrated that OLFM4 was significantly over-expressed in peripheral blood mononuclear cells (PBMCs) in pancreatic cancer patients, compared with a healthy control group [Yan H, et al. Hepatogastroenterology. 2011;58(109):1354-9]. In this study, we performed IHC analysis of OLFM4 expression using 80 pancreatic cancer tissues instead of peripheral blood samples. We then demonstrated a correlation between expression of OLFM4 and chemoresistance/poor prognosis in pancreatic cancer.

　We really appreciate this helpful comment. As indicated, we have modified the Discussion section (P31-33, lines 517-534, 537-539, 542-552, 562-567).

**Journal Requirement;**

1. When submitting your revision, we need you to address these additional requirements.

Please ensure that your manuscript meets PLOS ONE's style requirements, including those for file naming. The PLOS ONE style templates can be found at

http://www.journals.plos.org/plosone/s/file?id=wjVg/PLOSOne_formatting_sample_main_body.pdf and http://www.journals.plos.org/plosone/s/file?id=ba62/PLOSOne_formatting_sample_title_authors_affiliations.pdf

Answer:

We sincerely appreciate your helpful comment. I ensured that our manuscript met the style template of PLOS ONE’s requirement.

2. We note that one or more of the authors are employed by a commercial company: Noile-Immune Biotech, Inc

a) Please provide an amended Funding Statement declaring this commercial affiliation, as well as a statement regarding the Role of Funders in your study. If the funding organization did not play a role in the study design, data collection and analysis, decision to publish, or preparation of the manuscript and only provided financial support in the form of authors' salaries and/or research materials, please review your statements relating to the author contributions, and ensure you have specifically and accurately indicated the role(s) that these authors had in your study. You can update author roles in the Author Contributions section of the online submission form.

Please also include the following statement within your amended Funding Statement.“The funder provided support in the form of salaries for authors [insert relevant initials], but did not have any additional role in the study design, data collection and analysis, decision to publish, or preparation of the manuscript. The specific roles of these authors are articulated in the ‘author contributions’ section.”

If your commercial affiliation did play a role in your study, please state and explain this role within your updated Funding Statement.

b) Please also provide an updated Competing Interests Statement declaring this commercial affiliation along with any other relevant declarations relating to employment, consultancy, patents, products in development, or marketed products, etc.

Within your Competing Interests Statement, please confirm that this commercial affiliation does not alter your adherence to all PLOS ONE policies on sharing data and materials by including the following statement: "This does not alter our adherence to PLOS ONE policies on sharing data and materials.” (as detailed online in our guide for authors http://journals.plos.org/plosone/s/competing-interests). If this adherence statement is not accurate and there are restrictions on sharing of data and/or materials, please state these. Please note that we cannot proceed with consideration of your article until this information has been declared.

Please include both an updated Funding Statement and Competing Interests Statement in your cover letter. We will change the online submission form on your behalf.

Answer:

We sincerely appreciate your helpful comment. We provide an updated Funding Statement and Competing Interests Statement, as described below. Please change the online submission form.

Funding Statement;

This study was funded by Noile-Immune Biotech, Inc. All funding was performed as the collaborative research project and was only used to purchase the reagents and equipment needed for the experiment. The funder had no additional role in the study design, data collection and analysis, decision to publish, or preparation of the manuscript. The funder also provided support in the form of salaries to HI. The specific roles of these authors are articulated in the ‘author contributions’ section.

Competing Interests Statement;

The authors have read the journal’s policy and the authors of this paper have the following competing interests: HI is paid employees of Noile-Immune Biotech, Inc., clinical stage biotechnology company. There are no patents, products in development or marketed products associated with this research to declare. This does not alter our adherence to PLOS ONE policies on sharing data and materials.

3. We note that you are reporting an analysis of a microarray, next-generation sequencing, or deep sequencing data set. PLOS requires that authors comply with field-specific standards for preparation, recording, and deposition of data in repositories appropriate to their field. Please upload these data to a stable, public repository (such as ArrayExpress, Gene Expression Omnibus (GEO), DNA Data Bank of Japan (DDBJ), NCBI GenBank, NCBI Sequence Read Archive, or EMBL Nucleotide Sequence Database (ENA)). In your revised cover letter, please provide the relevant accession numbers that may be used to access these data. For a full list of recommended repositories, see http://journals.plos.org/plosone/s/data-availability#loc-omics or http://journals.plos.org/plosone/s/data-availability#loc-sequencing.

Answer:

We sincerely appreciate your helpful comment. We demonstrated the next-generation sequencing for the Patient-derived xenografts (PDX). The tumor tissues of PDXs were transplanted from pancreatic cancer patients for their surgical specimens. Exactly as you mentioned, NGS data should be uploaded to a public repository. However, the consents from patients have not been obtained to uploaded the whole genome sequence data to a public repository. Therefore, we thought that the data cannot be made public because of clinical tissue samples from patients. We appreciate your comment and apologize that we could not answer your requirement.

We believe that we have addressed the editor’s and reviewer’s comments and hope that revised manuscript is now suitable for publication in your journal. Thank you for your generous consideration.

Thank you and I look forward to hearing from you.

Sincerely yours,

Satoshi Wada, MD, PhD

Department of Clinical Diagnostic Oncology, Clinical Research Institute for Clinical Pharmacology and Therapeutics, Showa University, Tokyo 1578577, Japan

Tel: +81-3-3300-5257

Fax: +81-3-3300-1653

st-wada@med.showa-u.ac.jp
